# Analysis of Raised Feature Failures on 3D Printed Injection Moulds

**DOI:** 10.3390/polym13101541

**Published:** 2021-05-11

**Authors:** Anurag Bagalkot, Dirk Pons, Digby Symons, Don Clucas

**Affiliations:** Department of Mechanical Engineering, University of Canterbury, Private Bag 4800, Christchurch 8020, New Zealand; anurag.bagalkot@pg.canterbury.ac.nz (A.B.); digby.symons@canterbury.ac.nz (D.S.); don.clucas@canterbury.ac.nz (D.C.)

**Keywords:** rapid tooling, additive manufacturing, failure modes, injection moulding

## Abstract

Background: Polymer-based 3D Printed Injection Mould (3DIM) inserts are used as a cost-effective method for low volume injection moulding (50–500 parts). However, abrupt failure leading to a short tool life is a common shortcoming of 3DIM. Need: The underlying causes of raised feature failures on 3DIM are not well known. Failure is commonly attributed to bending or shearing of raised features on the tool. Understanding the causes may help in delaying the failure and increasing tool life. Approach: Tool failure was analysed from a first-principles perspective, using pressure and temperature fields as determined by mould flow simulation. Experimental results were also obtained for two types of tool material (Visijet M3-X and Digital ABS) with polycarbonate (Lexan 943A) as the part material. Findings: Results find against the idea that pin failure in 3DIM tools is caused by bending and shear failures induced by injection pressures. We also conclude that failure of raised features is not necessarily an abrupt failure as mentioned in the literature. Originality: The generally accepted explanation for the failure of raised features in 3DIM tooling is that injection pressures cause bending and shear failure. This paper disconfirms this notion on theoretical and experimental grounds.

## 1. Introduction

### 1.1. Injection Moulding (IM)

Injection moulding (IM) is one of the most commonly used polymer processing techniques, 35% by weight of all polymers are processed by IM [1]. Conventional IM tools are machined out of blocks of steel, aluminium or copper-beryllium alloys and the choice of mould material is dependent on factors such as the moulding material, the complexity of the part, the required life and the available budget [2]. The capital cost of an injection mould (tooling) is the largest cost component of an injection moulded part, followed by the moulding material and the processing costs [1]. Traditionally, IM has been a processing technique used for high volume production (>10,000 parts) and industries tend to amortize the high upfront cost of the tool over its expected life making it easier to justify the high upfront costs. To stay competitive, industries are looking to reduce the wastage of raw materials, shorten product lead times and eliminate the need for expensive tooling [3,4]. Master unit die (MUD) base or chase tooling was designed to reduce the cost of tooling [2]. A MUD base negates the need for expensive mould assemblies for each part and instead uses the same MUD base with different inserts (core and cavity) for different parts. A MUD base has pre-machined pockets into which mould inserts are fitted. Previously these mould inserts were conventionally machined. Improvements in additive manufacturing (AM) technology has led to the increased popularity of rapid tooling (RT) [5,6]. Industries are now using RT to produce mould inserts to be then fitted onto the traditional MUD bases. This has led to IM being a viable and cost-effective method of production even for short-run manufacturing (<500 parts) [4,7,8,9].

### 1.2. Rapid Tooling (RT)

Rapid Tooling (RT) is a process that uses AM (sometimes commonly referred to as 3D printing) techniques to build tools at low cost and short lead times compared to traditional manufacturing methods [10]. AM uses a layer-by-layer process to build parts and thus negates the need for complex conventional machining operations, direct labour and reduces wastage of raw materials [11]. RT techniques are usually classified as direct rapid tooling and indirect rapid tooling [12]. Direct RT involves the use of either metal or polymer AM systems to directly build tools, whereas indirect rapid tooling utilizes AM techniques to build master patterns which are then used to create the tool. IM tools are usually manufactured using direct rapid tooling techniques [13]. Rapid tools built using metal-based AM techniques are referred to as hard tooling and polymer-based tools are referred to as soft tooling in the IM industry [5]. At this current stage, the cost of metal AM systems and consumables are significantly higher than polymer-based AM systems. The most commonly used polymer AM techniques to build IM inserts are Stereolithography (SLA) and Material Jetting (MJ) [13].

### 1.3. Shortcomings of 3D Printed Injection Moulds (3DIM)

SLA systems have been used to manufacture soft tools for IM since the early 1990′s [4,14]. While the technique showed promise of cost and time savings, it did not garner widespread attention due to process and material limitations: issues such as dimension control, lack of material availability and poor mechanical and thermal properties of the material [15]. Selective laser sintering (SLS), fused deposition modelling (FDM) and material jetting are the other commonly researched polymer AM systems to produce 3DIM inserts [15,16,17]. SLS and FDM processes are widely used AM techniques in the RT industry but did not perform well in 3DIM applications due to their porous nature. The material used to produce 3DIM via SLA techniques had very poor mechanical and thermal properties compared to conventional tooling material (aluminium or steel) [6]. The inferior material properties led to abrupt failures of 3DIM generally due to thermal degradation and mechanical failure during the moulding process [15,18]. Failure was typically observed after several shots, while the moulding conditions reamined same. It was therefore suggested that there was a progressive deterioration of the 3DIM material [15]. The pressure exerted by the flowing polymer during the injection stage and packing stage was reported to cause deformation of the features on 3DIM leading to catastrophic failures [19]. 3DIM inserts tend to fail if the stresses created by the molten polymer exceed the yield strength of the tool material at elevated temperatures [20]. The surface roughness of 3DIM tools was not ideal due to the layered printing process and was reported as an important factor for 3DIM tool life [21]. In certain cases, surface smoothening of 3DIM was observed. The smoothening resulted in lower ejection forces in some cases, but in a majority of the cases resulted in excessive flashing [22].

The failure of 3DIM was often reported as a consequence of inferior material properties of the 3DIM. However, the IM process parameters were not taken into account and the usual process parameters that would be used with conventional IM tools were used with 3DIM as well. Process parameters were later determined to be a critical factor on the 3DIM tool life along with the design of 3DIM [23]. Parts moulded using 3DIM generally suffered from issues with dimensional stability, poor surface finish and quality due to thermal degradation and erosion of 3DIM [5,6]. The thermal conductivity of 3DIM materials is significantly lower than that of conventional tools, using the same cooling rate and time led to unformed parts in certain areas and excess shrinkage in certain areas of the part [23]. The processing temperature of IM polymer specified on the datasheet is for IM using a conventional tool. In the majority of cases, resin manufacturers specify a mould temperature which varies based on the type of resin but was generally found to be between 40–80 °C. Using these mould temperatures with 3DIM would lead to softening of the 3DIM as they are above the glass transition temperature (*T_g_*) of most 3DIM materials [24]. In our previous work, we also identified three major failure modes; surface failure, avulsion and feature failure [22].

### 1.4. Current State of the Art

The polymer AM industry has seen a lot of improvements over the last 10 years, new companies such as Formlabs™ have introduced significantly cheaper SLA systems such as the Form 3 [25] compared to the 3D Systems ™ Pro X [26] range of SLA machines and ProJet range of material jetting machines. In the Material Jetting space, the Polyjet line of systems from Stratasys ™ [27] has seen stiff competition from the like of Solidscape ™ [28] and Xjet ™ [29]. The new range of MJ machines have better accuracy and printing resolutions and require minimal post-processing. The new and improved AM systems have also led to the development of materials such as Digital ABS [24], Accura Bluestone [30], DSM PerFORM and Visjet M3-X. These materials have better mechanical and thermal properties compared to the materials used in the early SLA systems. The current improvements in the AM field provide an interesting challenge to use 3DIM to mould engineering and high-performance thermoplastics for low volume real-world applications. The main objective of this paper was to further investigate the feature failure modes in 3DIM.

### 1.5. Overview of the Paper 

Section 2 of this paper describes the theoretical and experimental methodology. Section 3 describes the theoretical and experimental results. Section 4 provides a detailed discussion of the results and highlights the scope for further research. In Section 5, we conclude the paper with suggestions on how to possibly improve 3DIM tool life.

## 2. Materials and Methods

### 2.1. Hypothesised Failure Mode: Pin Bending Due to Injection Pressure

Based on the literature review of Section 1, examination of failed 3DIM from past experiments and injection moulding experience, we hypothesize the following failure mechanism: 

Flexural and shear loads on raised features of 3DIM arise due to the pressure exerted by incoming polymer flow during the injection stage 

**Figure 1:**
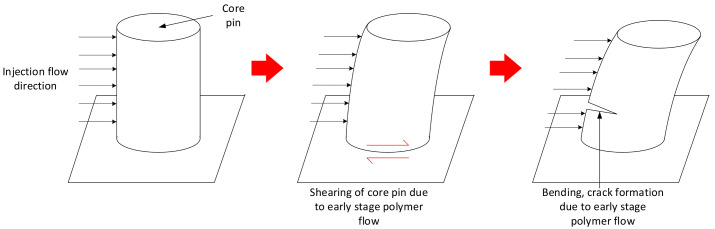
Graphical representation of hypothesized load case. This shows bending and shear of core pin due to incoming polymer flow during the injection stage.

We propose that, during the injection stage, the polymer melt exerts a force on the raised features of the tool, this force causes the raised features to bend and shear, per Figure 1. The bending and shear force cause the raised features to eventually break off. During the injection process, the temperature of the raised features also increases which also results in a lower yield strength, making it more susceptible to failure. This hypothesis is commonly supported in the literature [9,18].

### 2.2. Approach

To test this hypothesis, 3DIM moulds were designed, printed and used to injection mould until failure and the failure samples were analysed. Theoretical calculations were also performed.

#### 2.2.1. Design of a Representative Tool

**Figure 2:**
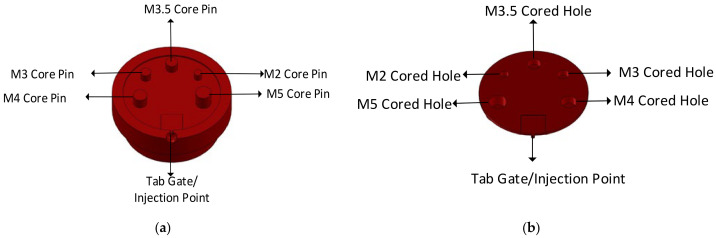
(**a**) Core side of 3DIM showing the raised features (core pins); (**b**) face of the part formed by the core insert.

**Figure 3:**
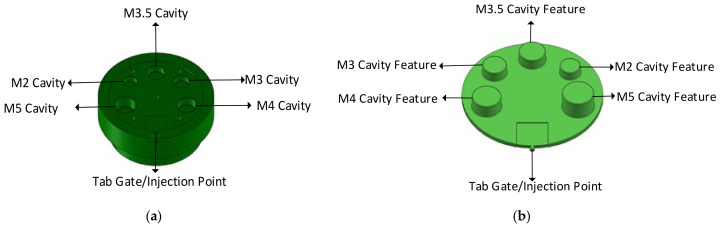
(**a**) Cavity side of 3DIM showing cored holes; (**b**)f of the part formed by cavity insert.

Several injection moulding tools at a plastic injection moulding firm (Talbot Technologies Ltd., New Zealand) were analysed to indicate the simplest and most commonly found raised features on a small-sized IM tool. The most commonly occurring raised features on IM tools were core pins which are used to create screw bosses or screw holes on the part. Following this analysis, a simple part geometry was developed. A flat circular plate design with 5 cored holes was used. The holes were designed to incorporate standard size M1–M5 threaded screw inserts. The sizing for the core holes was done according to specifications from threaded insert manufacturer SPIROL^®^ [31]. Different pin sizes were used to study the effect of geometry and aspect ratio on the tool life. SolidWorks^®^ 2019 [32] was used to design the part and the 3DIM. The specifications of the design are given in Table 1; refer to Figure 2 and Figure 3 for more details.

#### 2.2.2. Theoretical Analysis

Classical bending and shear stress formulae were applied to analyse the stress in raised features (pins) for the hypothesis. The input data required pressure and temperature of the melt and this was obtained from mould flow simulations. The simulation software used was Moldex3D^®^ R17 [33]. The input assumptions are described in the results. The pressure was then used to calculate the bending and shear stresses on the core pins.

#### 2.2.3. Empirical Testing

The 3DIM inserts were printed on two different MJ machines using two different resins; Table 1, below, gives details of the printing process. These 3DIM inserts were then used to injection mould parts until they failed using a polycarbonate resin with trade name Lexan-943A^®^ from SABIC^®^

The specifications of the additive manufacturing machine and the materials used for printing the 3DIM inserts are shown in Table 2.

**Figure 4:**
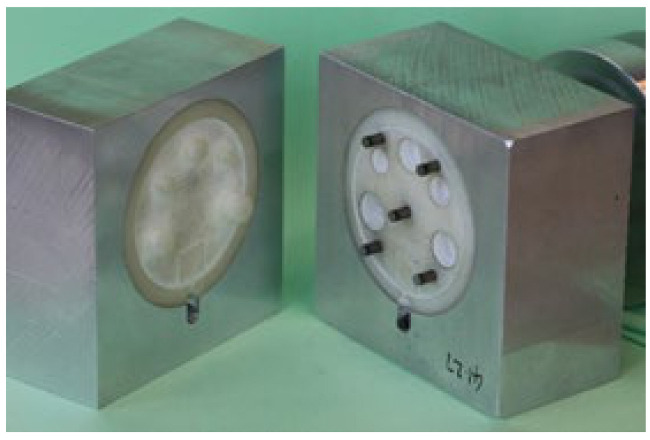
Core and cavity 3DIM inserts assembled inside a MUD base along with ejector pin assembly. The white parts are the 3DIM tools and the aluminium housings are the MUD base.

**Figure 5:**
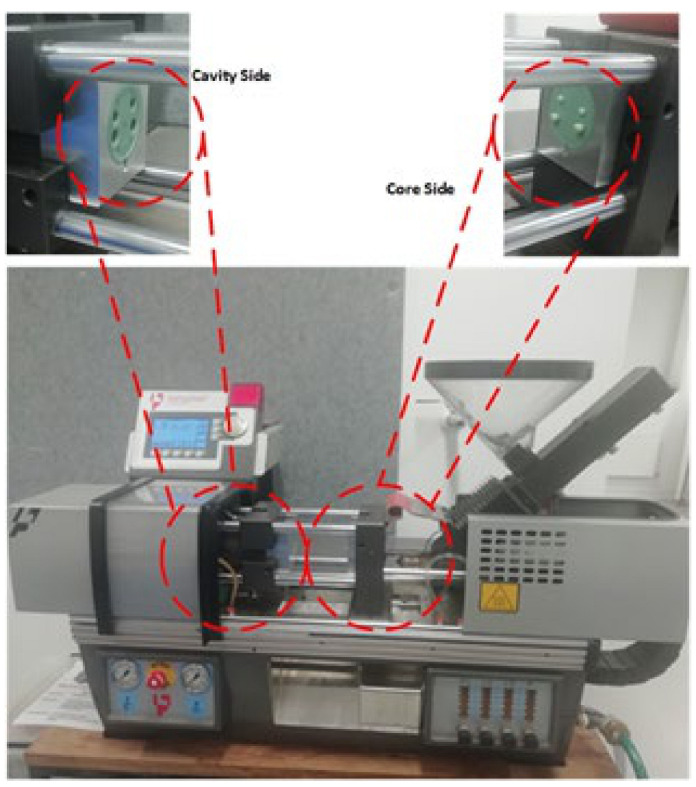
BabyPlast injection moulding machine with core and cavity 3DIM inserts assembled inside a MUD base and mounted onto the machine.

The 3DIM core and cavity inserts were assembled inside a master unit die (MUD) base as shown in Figure 4. The inserts were then mounted onto a Babyplast 10/12 IM machine as shown in Figure 5. The injection moulding material was dried at 80 °C for 6 h, as recommended by the material supplier, to remove any traces of absorbed moisture. Any unused material from an experiment was discarded as we wanted to avoid reheating the same batch of material before each experiment, which may lead to changes in the material properties.

A pilot tool similar to the ones used in the experiment was built and used to set the process parameters. This was done to optimize the injection pressure, cooling time and mould open time and to avoid abrupt failures of 3DIM during the experimentation. The injection moulding process parameters were set using the instruction mentioned in [23] and Table 3 shows the process parameters used during the experiments.

## 3. Results

### 3.1. Theoretical Results

#### 3.1.1. Pressure on the Pin 

**Figure 6:**
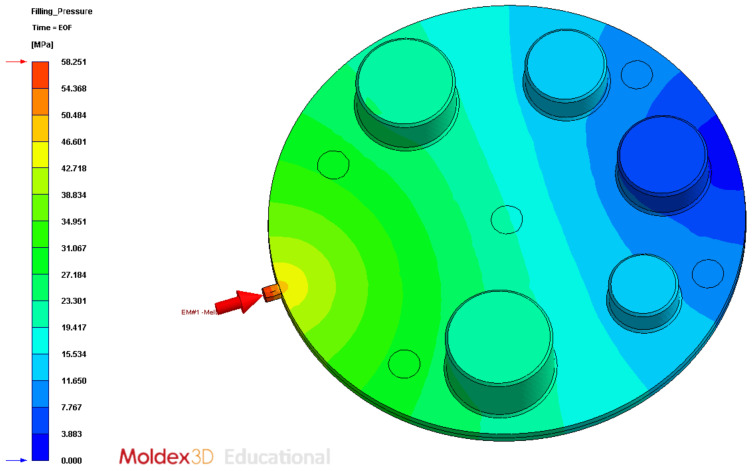
Pressure map at the end of filling cycle. The injection point is at left and the colours show the pressure of the molten polymer in the cavity at the end-of-fill stage.

**Figure 7:**
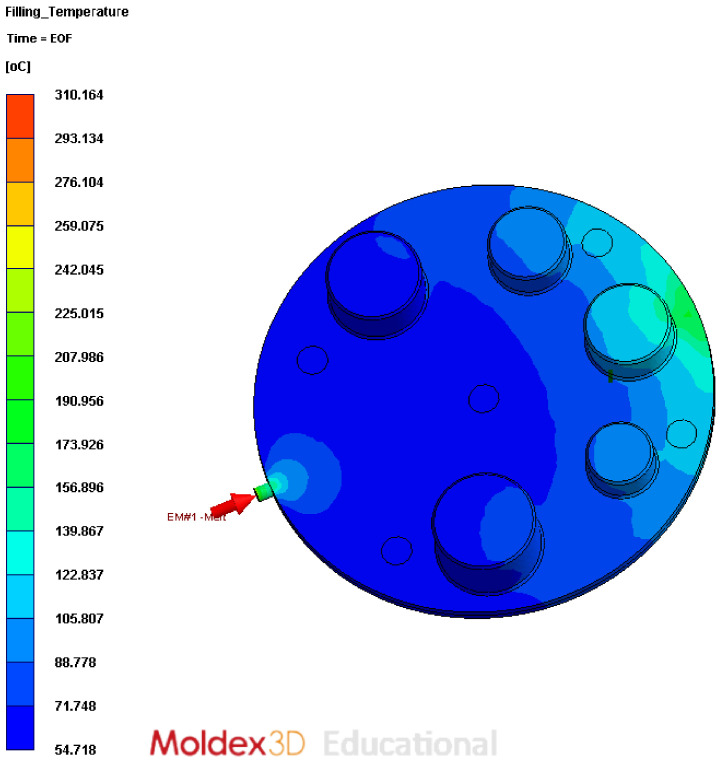
Temperature map at the end of filling cycle; the colours show the temperature of the polymer in the cavity at the end-of-fill stage.

The injection pressure refers to the force applied by the reciprocating screw inside the injection barrel to push the molten plastic resin into the mould cavity. The highest pressure experienced by the mould is at the gate (injection point) and, as the molten plastic flows through different sections of the mould, there is a loss in pressure in the flow due to drag and frictional effects. Figure 6 shows the pressure map and Figure 7 shows the temperature map close to the end of the filling cycle (98% filled cavity).

**Figure 8:**
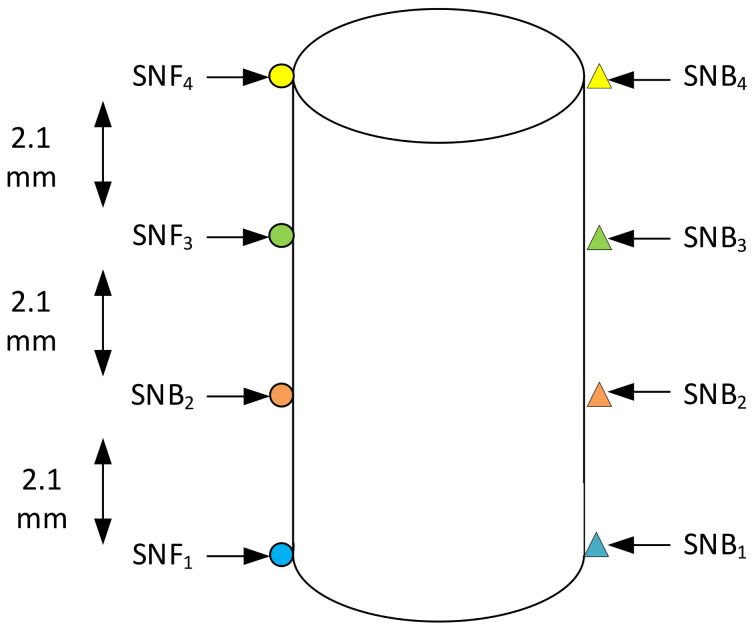
Location of sensor nodes on each of the core pins to determine the flow pressure using Moldex3D.

**Figure 9:**
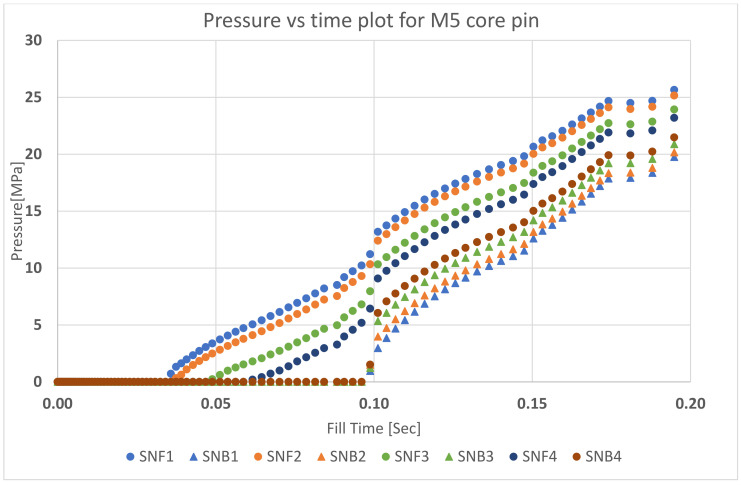
Pressure vs. time plot for M5 core pin. Sensor nodes are colour coded to depict the pressure difference between the front and back face of the core pin at different heights on the core pin.

The pressure and temperature on the on the tool were determined by simulation using Moldex3D. Several sensor nodes, as shown in Figure 8, were placed on the front and back face of each of the core pins to measure the injection pressure with respect to time. In Figure 9, the round dots (SNF_1_, SNF_2_, SNF_3_, SNF_4_) indicate the location of the first point (front face) of contact between the flowing polymer and the core pin and the triangles (SNB_1_, SNB_2_, SNB_3_, SNB_4_) indicate the location of the last point (back face) of contact between the flowing polymer and core pin. The sensor nodes were placed at a spacing of 2.1 mm starting from the base of the tool. Using these sensor nodes, the flow pressure over the whole injection moulding cycle with respect to time was measured; Figure 9 shows the graph for pressure vs. time for the M5 core pin.

**Figure 10:**
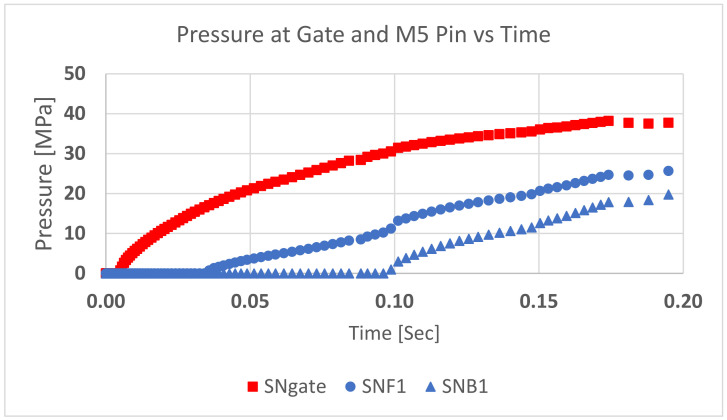
Pressure at the gate vs. M5 core pin. Pressure at the gate (SNgate) is higher than at the front face (SNF1). The pressure at the back face of core pin (SNB1) is less than the front face. The pressure drops along the flow length of the part.

The literature makes several severely simplifying assumptions in this area. First is the question of what pressure to use in any analysis. One approach uses the injection pressure itself, but as this is the pressure upstream of the gate, it is a severe overstatement of the actual pressure on the pins [9]. The pressure acting on the core pin is less than the pressure at the gate. In Figure 10, the pressure at the base of the M5 core pin is compared to the pressure just in front of the gate. The pressure at the gate is significantly higher than at the pins.

**Figure 11:**
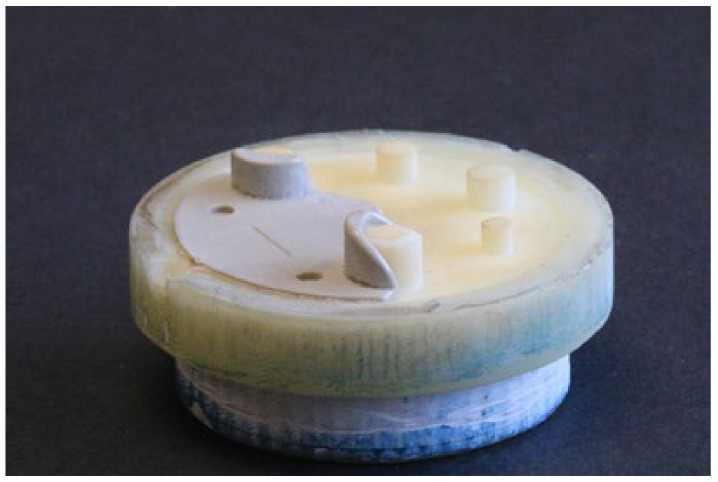
Frozen flow front from an incompletely moulded part; an incomplete part is assembled on the 3DIM to show the flow progression path.

**Figure 12:**
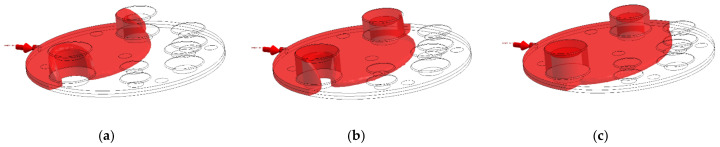
(**a**) Flow front when it covers the front face of the pin; (**b**) flow front moving over the pin; (**c**) flow front when the pin is fully surrounded by the melt.

The second question is where in the cycle to take the pressure. It appears that average cavity pressure at the end of the fill might have been used in some cases [34]. End of fill corresponds to the raised features being entirely surrounded by molten material and a condition of no-flow. Our results for this period are shown in Figure 6 and indicate that, while there is a large pressure gradient along the cavity as a whole, the gradient across any one pin is relatively low. At the end of the fill, the pressure on the front of a raised feature is counteracted by a similar pressure behind it. The time of highest pressure difference is during the fill, not at the end. The point of maximum pressure difference $\Delta P_{Pin}$ occurs when the melt front has covered the front of the pin and about to reach the rear of the pin, which is at the time $t^{*}$. The actual flow pattern in molds is shown in Figure 11. The physical flow of the material over the pin is relatively complex and the progression is simulated and shown in Figure 12.

**Figure 13:**
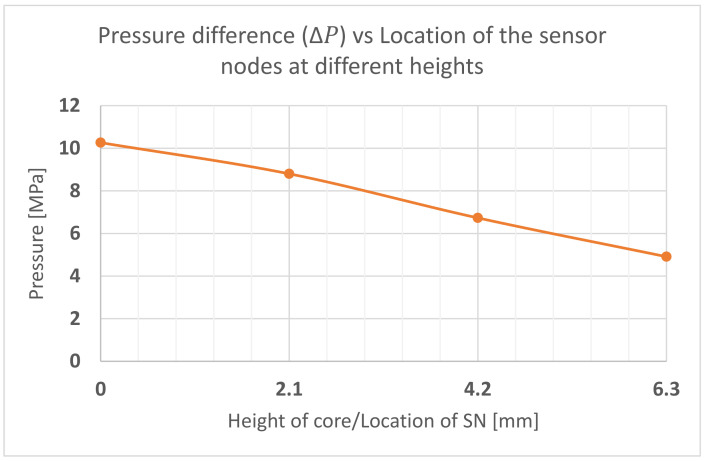
Pressure difference vs. the height of the core pin. The sensor nodes at the front and back were used to simulate the pressure difference. See Figure 9 for sensor node configuration.

The third question is what pressure distribution exists on the pin. Some have considered the pressure to be uniformly distributed over the front surface [9]. Our approach is to determine the pressure by using sensor nodes. The shape of the pressure distribution is determined by the extraction of the pressure points at $t^{*}$ during which $\Delta P_{Pin}$ is the highest. $\Delta P_{Pin}$ refers to the difference in pressure measured between the front (SNF_i_ sensor nodes) and back (SNB_i_ sensor nodes) of the core pin at any given time. $\Delta P_{Pin}$, for each set of sensor nodes at different heights, was calculated to obtain the pressure profile on the core pin. For example, for pin M5, the maximum Δ_pinSN1_ between the front (location SNF_1_) and back of the pin (SNB_1_) occurs at a time $t_{M5}^{*}=0.098\;s$. The graph in Figure 13 shows the $\Delta P_{Pin}$ pressure distribution for the M5 core pin at $t^{*}=0.098\;s$. The distribution may be approximated by a straight line, i.e., a linearly decreasing load with the highest pressure at the base of the pin and lower pressure at the top of the pin. 

**Figure 14:**
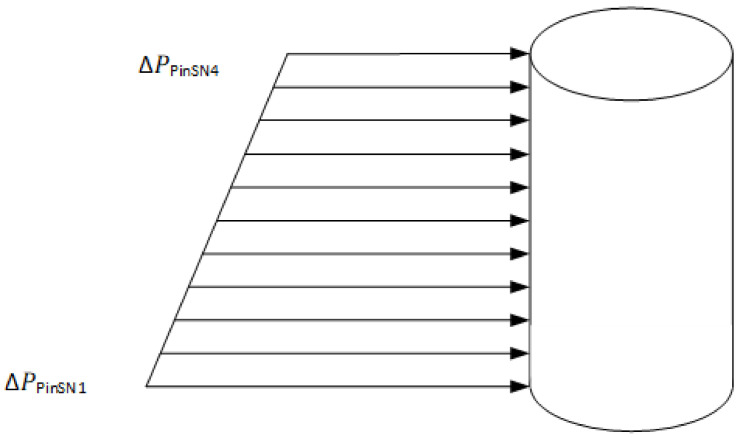
Linearly decreasing pressure profile on the core pin.

The pressure distribution on the core pin can therefore be simplified to a linearly varying load on a cantilever beam as shown in Figure 14.

#### 3.1.2. Bending Stress

**Figure 15:**
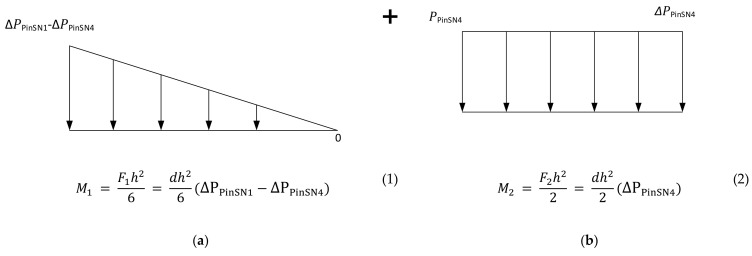
(**a**) Linearly decreasing load profile; (**b**) uniformly distributed load profile.

Bending stress on the core pin is highest when the pressure exerted by the melt front is the highest: for each of the core pins, $t^{*}$ at which $\Delta P_{Pin}$ is highest was measured from the mould flow analysis. The bending moment for the load profile shown in Figure 14 is solved as a combination of a uniformly distributed pressure and a linearly varying pressure as shown in Figure 15. The force *F* per unit length of pin is the pressure times the width of the pin (*d*). Variables are described in Table 4.

The bending stress on the pins $\sigma_{max}$ is given by the sum of the above moments, applied to a pin of circular cross-section:

(3) $$\sigma_{max}=\frac{My}{I}=\frac{M}{\frac{\pi d^{3}}{32}}=\frac{\frac{1}{2}h^{2}d\left(\frac{2}{3}{\Delta P}_{\mathrm{PinSN}4}+\frac{1}{3}{\Delta P}_{\mathrm{PinSN}1}\right)}{\frac{\pi d^{3}}{32}}$$

The results of this analysis are shown below. These are merely selected results showing the peak bending stress on each pin and the time at which it arises in the filling cycle.

#### 3.1.3. Shear Stress

The shear stress variables are identified in Table 5. The average shear stress in the pin cross-section is given by:

(4) $$\tau_{max}=\frac{S}{A}$$

The total shear force *F* at the base of the pin for the loading case shown in Figure 14:

(5) $$S=\frac{F_{1}h}{2}+F_{2}h$$

Therefore, the shear stress $\tau_{max}$ applied to a pin of circular cross-section:

(6) $$\tau_{max}\;=\;\frac{S}{\frac{1}{4}\pi d^{2}}\;=\;\frac{\frac{dh}{2}\left(\Delta_{\mathrm{PinSN}4}+\Delta_{\mathrm{PinSN}1}\right)}{\frac{\pi d^{2}}{4}}$$

The results of this analysis are shown in Table 6. These are merely selected results showing the peak shear stress on each pin and the time at which it arises in the filling cycle.

**Figure 16:**
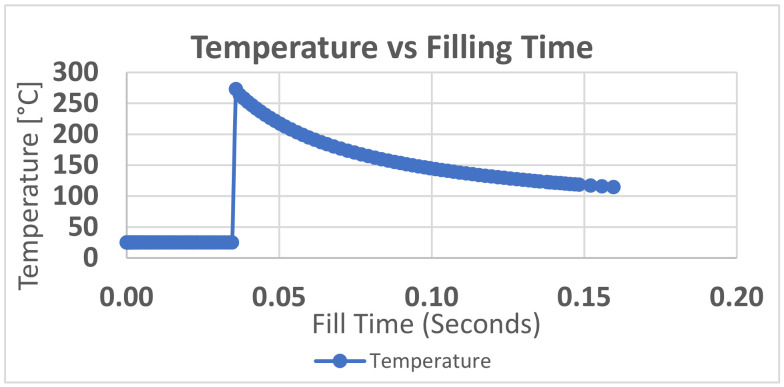
Temperature vs. fill time at SNF1 on M5 core pin from Moldex3D; the temperature of the pin suddenly increases as the molten polymer touches it and it starts to gradually cool over the injection cycle.

Nominally, it appears that the bending stress and shear stress does not exceed the yield strength of the tool material. The yield strength of the material is 42.5 MPa at room temperature, as mentioned in the material data sheets. However, this is simplistic, as the yield strength is a function of temperature and the operating temperature of the tool is higher than room temperature. The surface temperature of the tool was around 75–80 °C when measured at the end of moulding cycle. This temperature is the not the highest operating temperature, as this was measured at the end of cycle when the mould opens. Moldex3D simulations show that the highest surface temperature of the tool was about 288 °C during the injection cycle. The surface temperature variation over the injection cycle is shown in Figure 16. 

**Figure 17:**
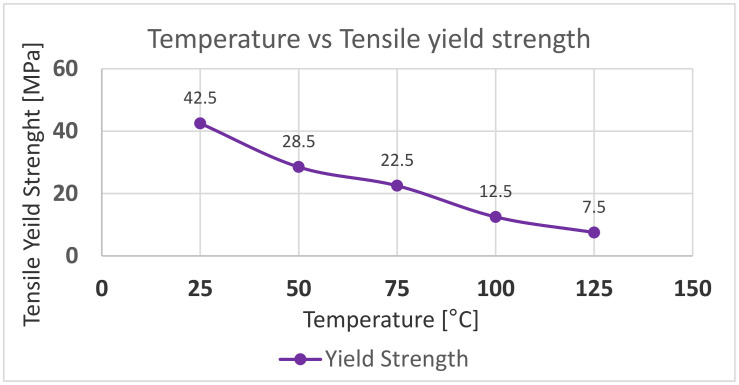
Tensile yield strength vs. temperature as reported in the literature.

To determine if the bending and shear stresses were causing the pins to fail, we needed to compare it to the yield strength of the tool material at operating temperatures. The data for yield strength at elevated temperatures were not provided in the data sheets and we did not have the equipment available to run tensile tests at temperatures above 50 °C. Hence, we used the data from the literature to obtain the material properties as shown in Figure 17 [5]. To verify the data in the literature we also performed tensile tests using the same standards and ran tests at 25 °C and 50 °C. The tensile strength at these temperatures matched with the values reported in the literature. The drop in yield strength around 50 °C is explained by the fact that the *glass transition temperature (T_g_)* is reported to be between 47–53 °C.

Figure 17 shows the yield strength of the material dropping significantly at elevated temperature. Comparing the results in Table 6 with the yield strength of the tool material (7.5 MPa) at operating temperature indicates that the core pins would fail due to the bending and shear stresses. However, due to the short cycle time and the tool material being an insulator, we suspected that the heat does not penetrate the central parts of the core pins and that the temperature distribution would not be uniform. 

**Figure 18:**
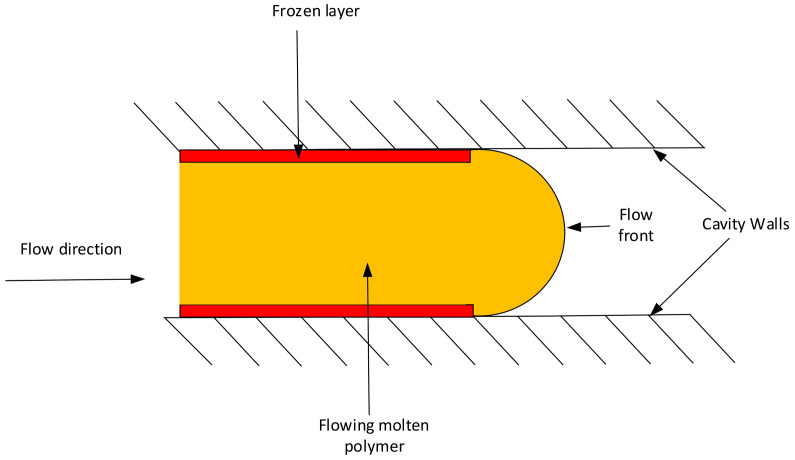
Flow of molten polymer inside the cavity showing frozen layer.

The typical flow of polymer inside the cavity is shown in Figure 18, the molten polymer freezes as soon as it touches the core and cavity walls and forms a frozen layer. The average frozen layer ratio obtained from Moldex3D was 6% (two frozen layers relative to the whole thickness).

To address the above problem, a transient thermal analysis using ANSYS WORKBENCH 2019 R3 was performed on the core pins to determine the heat conduction and temperature distribution. A worst-case scenario was considered, wherein all the heat at the surface would be transferred to the core pins, without heat loss. In practice, the polymer of the part would also have low thermal conductivity and, hence, not all its heat would be experienced by the core pin. 

**Figure 19:**
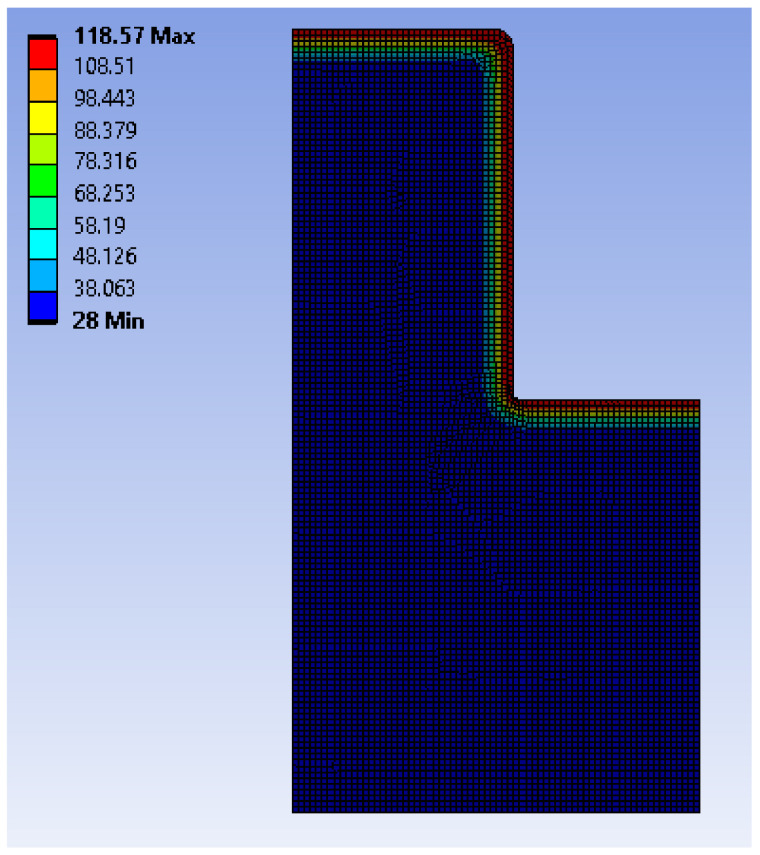
Temperature gradient on the M5 core pin; the pin is shown as a 2D axis symmetrical model.

**Figure 20:**
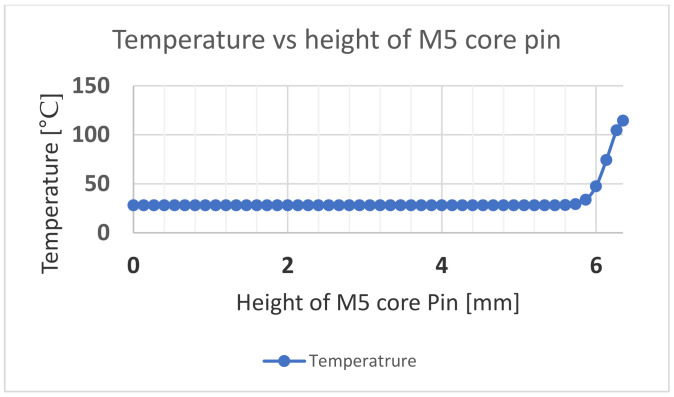
Temperature vs. the height of the core pin measured at a distance of 3.58 mm from the outer surface.

**Figure 21:**
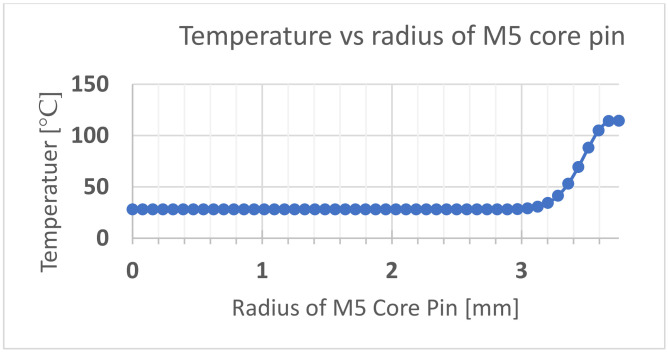
Temperature vs. the radius of the core pin measured at a height of 3.75 mm from the base of the pin.

The initial ambient temperature of the core pin was set at 28 °C. A 0.1 mm frozen layer was modelled around the pin based on the frozen layer ratio of 6% on a 1.5 mm thick part. The temperature of this frozen layer (boundary condition) was the simulated temperature at the surface of the core pin as a time-dependent variable based on the flow simulation results from Moldex3D. The time frame considered was from the start to the end of the injection cycle (0.2 s). After this period, the bending and shear forces are zero during the cooling stage, even if the pin does continue to absorb more heat. Figure 19 shows the thermal distribution across the pin at the end of the injection cycle. It is observed that the heat does not penetrate the central locations of the pin. Figure 20 shows the temperature distribution with respect to height measured at a distance of 3.58 mm from the outside surface. Figure 21 shows the temperature distribution with respect to the radius of the core pin measured at a height of 3.175 mm from the base of the pin.

Since the temperature distribution on the core pin was not uniform, the yield strength of the material is also non-uniform across the section of the pin and also changes with time. We conservatively assumed that any part of the core pin that was over 30 °C contributed nothing to structural strength due to low yield strength at elevated temperatures. From the pressure perspective, the whole pin geometry was used but for the load-bearing capacity only the central cooler volume. The depth at which the temperature of the core pin raised above ambient temperature was noted and this was used to determine a new effective diameter ($d_{eff}$) of the core pin. For example, only up to a depth of 0.55 mm was the material at the M5 pin core pin at higher than ambient temperature. The transient thermal analysis was repeated for the smallest pin (M2), which is the most conservative case for reduction in diameter. This reduction was then applied to the other core pins M2–M4. Subtracting this value from the original radius of the pin we get the new effective diameter. Hence, the stress Equations (3) and (6) become as follows:

- for bending:
  (7) $$\sigma_{max}=\frac{My}{I}=\frac{M}{\frac{\pi d_{eff}^{3}}{32}}=\frac{\frac{1}{2}h^{2}d\left(\frac{2}{3}{\Delta P}_{\mathrm{PinSN}4}+\frac{1}{3}{\Delta P}_{\mathrm{PinSN}1}\right)}{\frac{\pi d_{eff}^{3}}{32}}$$
- for shear:
  (8) $$\tau_{max}=\frac{F}{\frac{1}{4}\pi d_{eff}{}^{2}}=\frac{\frac{dh}{2}\left(\Delta_{\mathrm{PinSN}4}+\Delta_{\mathrm{PinSN}1}\right)}{\frac{\pi d_{eff}{}^{2}}{4}}$$

The theoretical analysis was repeated using the stress Equations (7) and (8). Results are shown in Table 7. Bending and shear stresses on the core pin with the effective diameter and yield strength of the tool material. The bending and shear stresses on the core pin do not exceed the yield strength of the material. We can conclude that, even with conservative assumptions, the failure of core pins is not due to the stresses created by the flowing polymer. These results also agree with the experimental results shown in the next section.

#### 3.1.4. Rejection of the Hypothesis

We conclude that, on theoretical grounds, even with conservative assumptions, the failure of core pins (raised features) is not due to stresses created by the flowing polymer. The results shows that the bending and shear stresses are below the yield strength of material, even allowing for the thermal degradation of outer layers of the pin. In the next section, we validate this conclusion experimentally.

### 3.2. Experimental Validation 

#### 3.2.1. 3DIM Insert A (Visijet M3-X)

**Figure 22:**
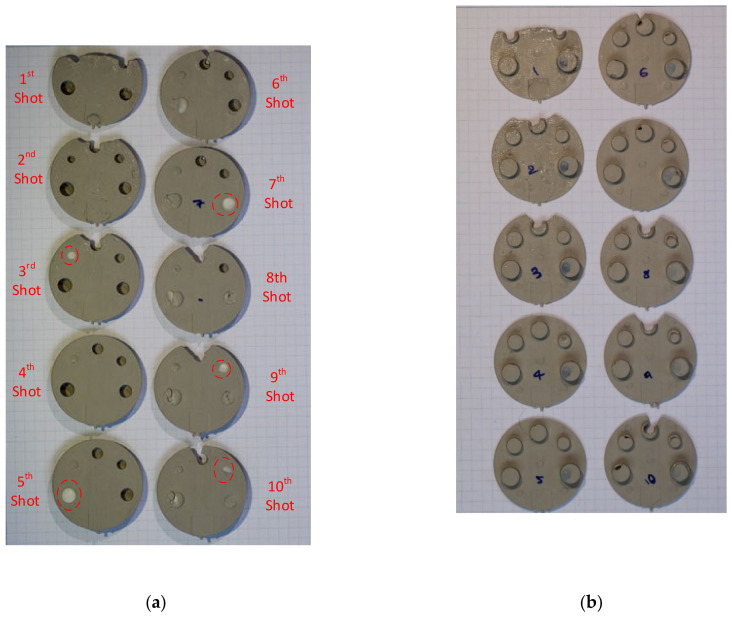
(**a**) Core face of the moulded part showing signs of progressive deterioration; (**b**) cavity side of the moulded showing no signs of progressive deterioration (moulded using insert A). The red circle highlights the area of interest and shows deterioration of the core pin (broken bits of core pin) after each shot.

The Visijet tool had a draft angle of 1.5 degrees on both the core and cavity insert. Multiple shots were injected and the results are shown in Figure 22, Figure 22a for the core face of part and Figure 22b for the cavity face of the part. The tool material was white in colour and broken fragments thereof are evident in the parts. The following comments are made about the sequence, primarily with reference to the Figure 22a images:

Shots 1–3: The moulding run commenced with a lower shot volume of 75% and incrementally increased to 85% and 95% for 2nd and 3rd shot. The part shows incomplete fill at the furthest distance from the gate around the M3.5 core pin. Note that the gate is at the bottom of the part on the opposite end of M3.5 core pin.
 Shot 3: M2 core pin failed. This was the most slender and shortest of the pins.
 Shot 4: Full shot, but the M2 hole is solid and filled with injection material for the rest of the sequence (M2 core pin from the tool is broken in previous shot).
 Shot 5: Full shot, M5 core pin initial failure.
 Shot 6: M5 core pin has a further failure. Incomplete fill arises at the furthest distance from the gate, around the M3.5 core pin. For explanation of this phenomenon, see below.
 Shot 7: M4 core pin fails. The M5 hole is solid and filled with injection material for the rest of the sequence. Incomplete fill apparent around the M3.5 core pin: see corresponding cavity face in Figure 22b.
 Shot 8: M4 hole is solid and filled with injection material for the rest of the sequence. M5 core pin has a further small avulsion failure. Incomplete fill arises at the furthest distance from the gate.
 Shot 9: M3 core pin fails. Incomplete fill arises at the furthest distance from the gate.
  Shot 10: M3 core pin has a further failure. Incomplete fill arises at the furthest distance from the gate.

**Figure 23:**
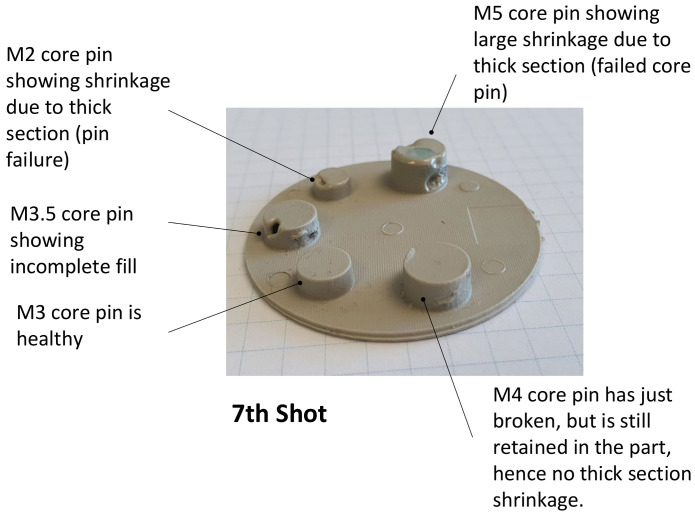
Part 7, showing a number of different defects on the cavity face.

Explanation for incomplete fill around the M3.5 core pin from shots 6–0:

The incomplete fill in shots 1–3 shown in Figure 22 is due to the deliberate use of lower shot volume at the start of the moulding cycle. This is common practice in the industry and is intended to avoid safety hazards (e.g., blockages and pressure drops). Incomplete fills from shot 6 onwards have a different reasoning. The cause is attributed to the diversion of shot volume into the space vacated by the broken M2 core pin and subsequently the broken M5 core pins. On the failure of the M5 core pin, the area which was supposed to be a cored hole with 1.5 mm wall thickness is replaced with a cylinder (thick section) of diameter 7.16 mm. Consequently, there is less volume available for elsewhere in the part and the deficit is apparent at the furthest distance from the gate, which is around the M3.5 core pin. This not only causes short fills, but also causes non uniform flow and shrinkage due to thick sections. The deficit does not manifest in exactly the same way each time. This variability is evident to some extent in Figure 22a and more so in Figure 22b. A close-up image of Shot 7 cavity face is shown in Figure 23, with an explanation of the various defects.

It appears that shot 10 shows higher filling compared to shots 7–9, when considering the V notch formed around the M3.5 pin. This is somewhat anomalous. Inspection of the cavity face of the parts 7–10 shows a number of small differences in the shapes of the features. The parts were also weighed using a scale and did not show a huge variation that would explain the higher fill percentage. In summary, as the core pins deteriorate and geometry is lost off the tool, the shot volume is consumed to fill the new voids. Since the shot volume is fixed, this means less material is available for elsewhere and the shortfall is most apparent on the last flow point. This corresponds to M3.5 core pin and surrounding area.

#### 3.2.2. 3DIM Insert B (Digital ABS)

3DIM inserts B and C used a different material which was green in colour. This has the additional benefit of making it easier to photograph the deterioration of core pins because of the improved optical contrast. 

This set of inserts had a draft angle of 1.5 degrees on both the core and cavity features. No damage was seen on the cavity side of the part.

**Figure 24:**
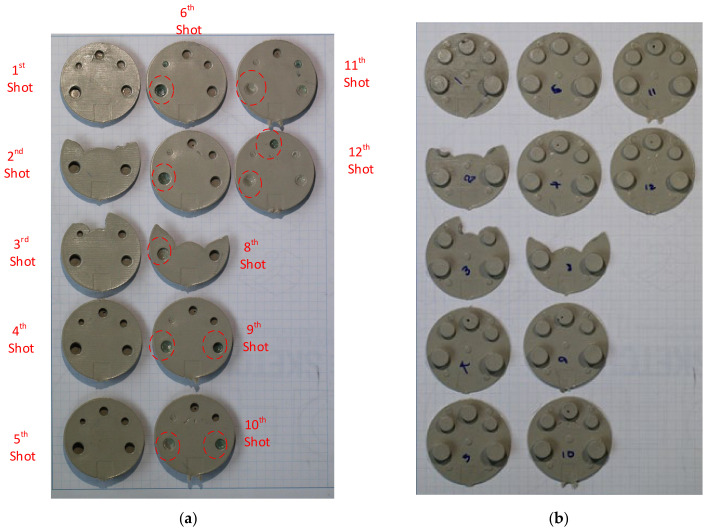
(**a**) Core face of the moulded part showing signs of progressive deterioration; (**b**) cavity side of the moulded showing no signs of progressive deterioration (moulded using insert B). The red circles highlight the areas of interest and show deterioration of the core pin (broken bits of core pin) after each shot.

The failure sequence of the core pins is shown in Figure 24 and was as follows:

- 6th shot—top part of the M2 core pin
- 6th shot—top part of the M5 core pin
- 7th shot—top part of the M5 core pin
- 9th shot—M4 and M5 core pin
- 11th shot—M3 core pin
- 12th shot—top part of the M3.5 core pin

**Figure 25:**
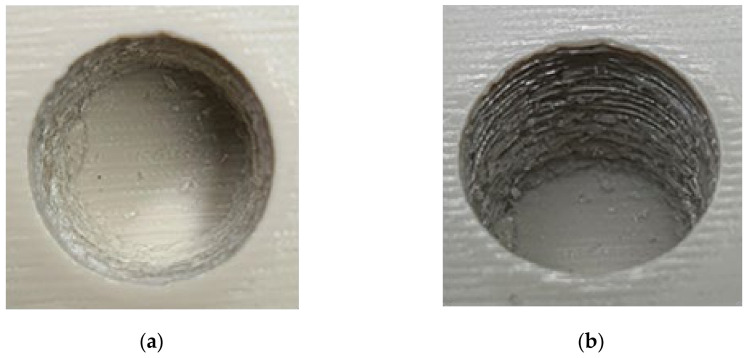
(**a**) Top view of M5 core on the pin on 5th part showing no signs of deterioration; (**b**) orthographic view of M5 core hole on 5th part showing no signs of deterioration.

**Figure 26:**
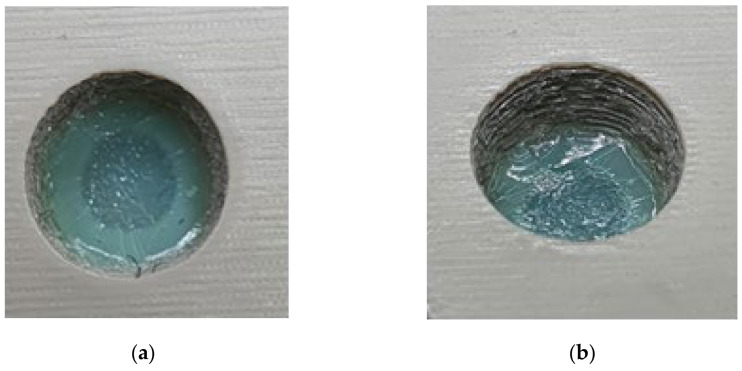
(**a**) Top view of M5 core on pin 6th part showing a small chunk of green 3DIM material of core pin stuck inside; (**b**) orthographic view of M5 core hole on 6th part showing a small chunk of green 3DIM material of core pin stuck inside.

**Figure 27:**
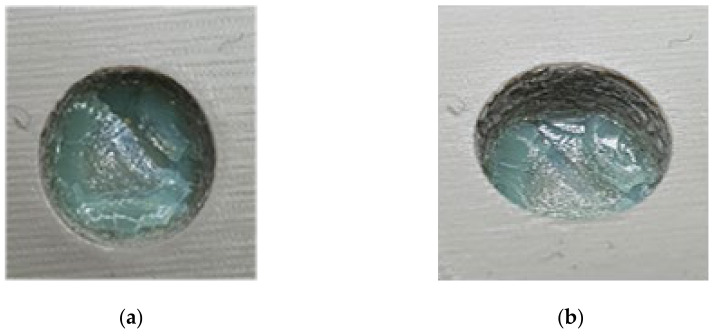
(**a**) Top view of M5 core on pin 8th part showing a small chunk of green 3DIM material of core pin stuck inside; (**b**) orthographic view of M5 core hole on 8th part showing a small chunk of green 3DIM material of core pin stuck inside.

**Figure 28:**
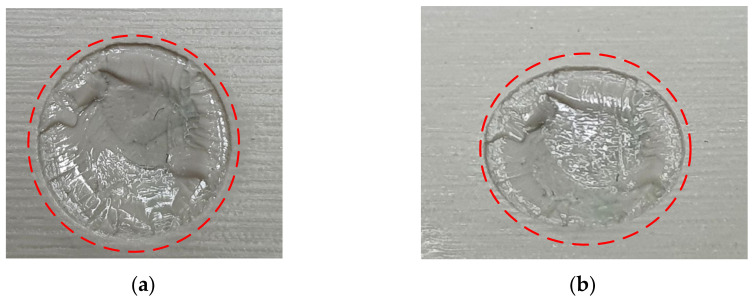
(**a**) Top view of M5 core on pin 12th part showing a small chunk of green 3DIM material of core pin stuck inside; (**b**) orthographic view of M5 core hole on 12th part showing a small chunk of green 3DIM material of core pin stuck inside.

**Figure 29:**
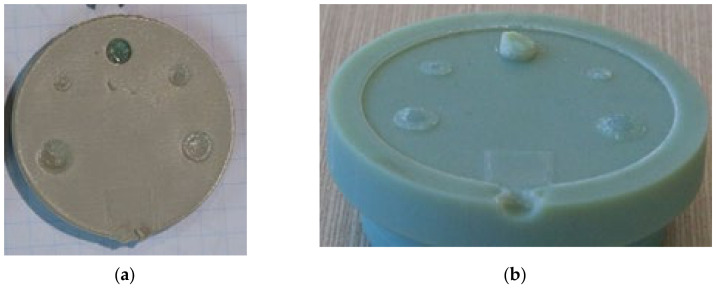
(**a**) 12th moulded part showing defects (**b**) State of core pins (broken) on the 3DIM after 12 shots. The moulded parts have no cored holes as the core pins on the tool are completely broken.

As seen in Figure 24, the top half of the M5 core pin broke on the 6th shot. This resulted in more molten material accumulating in that area creating thick sections, which leads to more shrinking and eventually causes the whole pin to be pulled off. As the whole pin has been pulled off on the 7th shot, the section which was supposed to be a hole, is now a thick section. The failures of core pins in both 3DIM inserts A and B were progressive. Initially, it started with chipping of small bits from the top of the core pin and progressively deteriorating to chipping out bigger chunks of the pin after each moulding shot and then eventually leading to complete failure of the pin. To record this progressive deterioration, we photographed each of the cored-out holes on the injection moulded part individually. The progressive deterioration of the core pin would result in a progressive reduction of the cored hole size and this is shown in the sequence of images from Figure 25, Figure 26, Figure 27, Figure 28 and Figure 29. In Figure 25, the top and orthographic view of the M5 cored hole is shown; the hole is completely formed here. This is the part moulded before the first sign of deterioration. In Figure 26, the first chipping of the 3DIM core pin can be seen stuck inside the 6th moulded part. The green piece of material inside the mould is the top part of the core pin which has been chipped and stuck inside the part.

**Figure 30:**
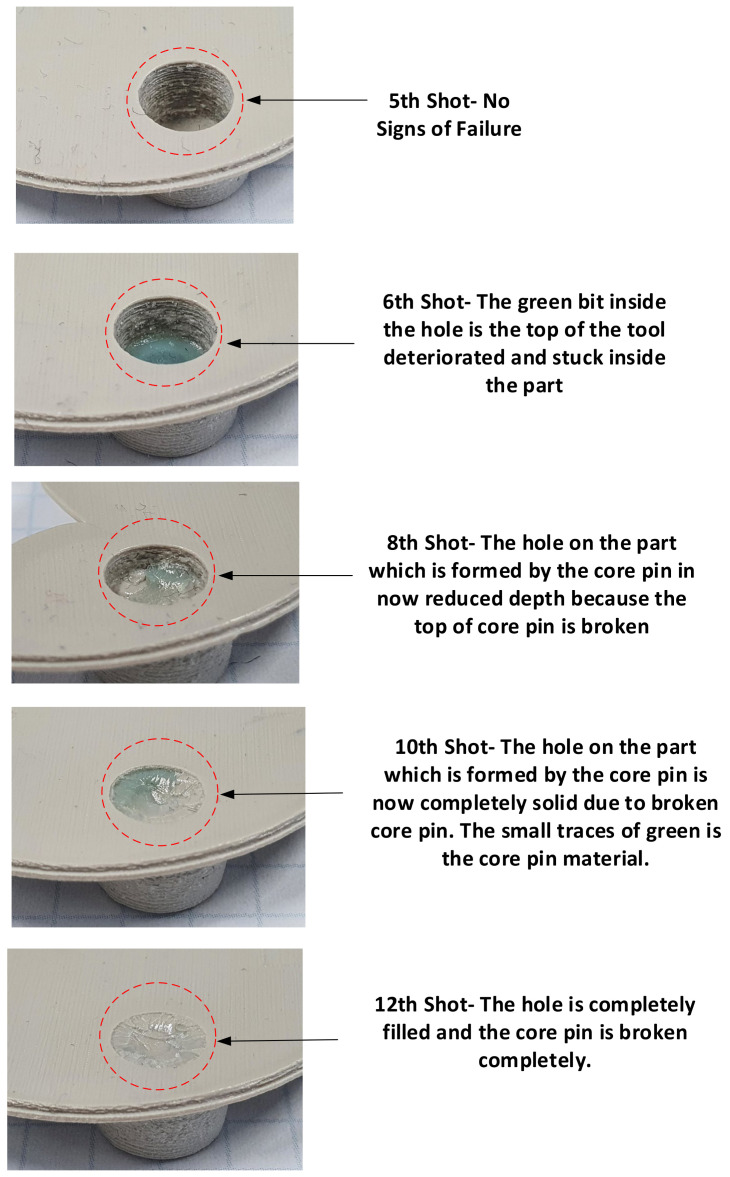
Progressive reduction in depth of the M5 core hole as a result of deterioration of the M5 core pin.

The complete progressive deterioration of the core pin can be seen in Figure 30 and to validate this we also measured the hole size after each shot. When the core pin starts deteriorating, the hole size on the part should reduce and this was validated and is shown in Table 8.

#### 3.2.3. 3DIM Insert C (Digital ABS)

In the previous experiments, the farthest pin from the gate which is the M3.5 pin was the least damaged. Hence, we decided to remove the pin from this set of 3DIM inserts to reduce the complexity and see if shrinkage and cooling had any effect on the failure of core pins. The same process parameters used in previous experiments were used and the shot size was reduced by the required quantity. 

**Figure 31:**
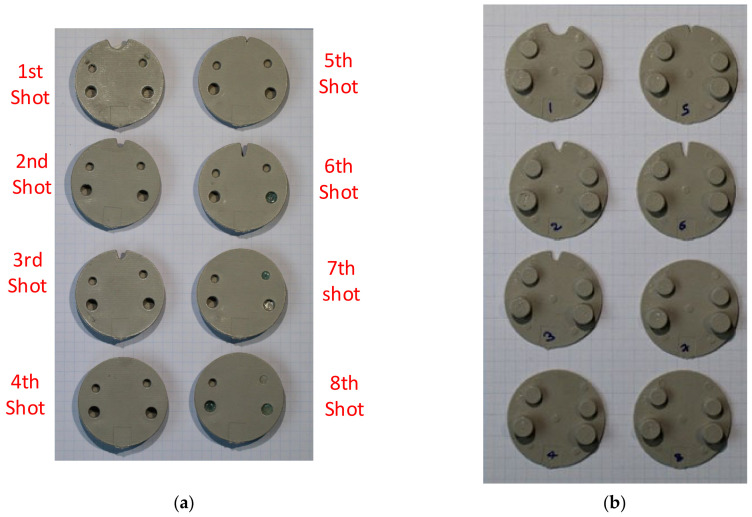
(**a**) Core face of the moulded part showing signs of progressive deterioration; (**b**) cavity side of the moulded showing no signs of progressive deterioration (moulded using insert C).

The failure sequence of the core pins is highlighted in Figure 31 and was as follows:

- 6th shot—top part of the M4 core pin.
- 7th shot—top part of the M2 core pin.
- 8th shot—top part of the M5 core pin

3DIM insert C also showed progressive deterioration and eventual failure of the core pins. However, the failure sequence of the core pins was different from inserts A and B. Initial experiments using 3DIM inserts A and B showed that even though there is progressive deterioration and failure, the smaller M2 and M3 core pins were the ones that deteriorated and failed initially leading to the failure of M4 and M5 inserts. However, in the case of 3DIM insert C, the bigger M5 insert started to fail first leading to the failure of the smaller core pins.

## 4. Discussion

### 4.1. Findings

As shown in the theoretical results section, the heat does not penetrate to the centre of the core pin, thus resulting in only the outer surface of the tool reaching higher temperature and losing its strength. Further experimental results also showed that the core pins started deteriorating at the outer surface and failing. The failure of raised features was not due to bending or shear, they followed a more progressive deterioration. 

Failure of raised pin features was not observed to occur at the base of the pin which would be expected in bending and shear failures. Rather, there was a progressive deterioration of the tip of the pin and around the surface, whereby material was chipped off in layers and was stuck inside the tool. The combination of bending and shear at the root of the pin appears to be the highest stress during the injection stage, but this does not result in the failure of the pin. We, therefore, reject the hypothesis that pin failure in 3DIM tools is caused by bending and shear failures induced by injection pressures. We also conclude that failure of raised features is not necessarily an abrupt failure, as mentioned in the literature. 

A comparison of 3DIM tooling vs. conventional aluminium/steel tooling is shown in Table 9.

Based on the current findings and previous work [22], the failure of raised features for 3DIM is classified as shown in Table 10.

**Figure 32:**
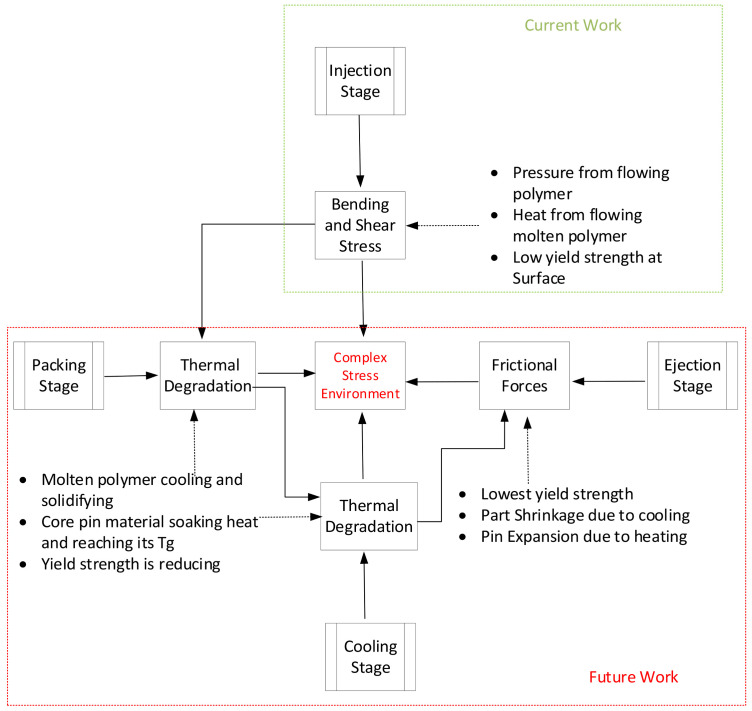
Potential causes of failure of core pin.

Based on the findings, we propose a new theory: that the failure of raised features is due to the combined effect of stresses developed during the injection stage, thermal degradation during the cooling stage, with shrinkage and frictional forces during the ejection stage of injection moulding. Figure 32 shows the potential causes of failure.

### 4.2. Limitations of the Research

The arrangement of the core pins on the part will affect the flow of resin during the injection stage and the temperature distribution during the packing and cooling stages. An ideal solution would have been to use direct sprue gate at the centre of the part such that the distance of each core pin would be uniform from the melt flow entrance. In our experiments, the gate was located at the edge of the part and the distance between the gate and each of the core pin was not uniform. This was primarily done to fit the 3DIM tool inside the platen of the IM machine. The layout of the pins was not the primary purpose of this study, but in general, it is expected that a pin closer to the gate would start degrading earlier than the pins located farther from the injection point.

### 4.3. Implications for Future Research

The injection stage during the moulding process is short (0.2 s). This is a short time for heat transfer between molten polymer and core pin. While the core pin is at the ambient temperature, the bending and shear stresses developed due to flow pressure are lower than the yield strength of the material. However, the core pin is still absorbing heat during the packing stage (0.2 s), cooling stage (45 s) and until the mould opens. The average surface temperature of the core pins when measured at ejection using a non-contact thermometer was around 75 °C. We suspect that the long cooling time would result in the central locations of the core pin reaching higher than ambient temperatures.

## 5. Conclusions

This paper evaluated hypothesis that raised features fail due to bending and shear stress created by injection pressure. This was evaluated on theoretical and experimental grounds. Conservative assumptions were made about the magnitude of injection pressure, temperature distribution and heat transfer across the core pins. The theoretical analysis was supported by pressure and thermal data obtained from flow analysis using Moldex3D. Theoretical results show that the bending stress was below the yield strength of the tool material even at elevated operating temperatures. Hence, the hypothesis of bending failure was rejected on theoretical grounds. This was then validated experimentally, by examining two different types of 3DIM material and two core pin configurations on the part. Qualitative examination of the failure modes using optical microscopy showed that the failure occurred as chipping of the edges of the raised features, not cracking at the root (where bending would act). Hence, on both theoretical and experimental grounds, we reject the hypothesis that raised features in 3DIM tools fail primarily due to bending stress from the injection pressure or the localized pressure.

## Figures and Tables

**Table 1:** Dimensions of the core pin.

Name	Diameter (mm)	Height (mm)	Aspect Ratio	Distance from Gate (mm)
M2 Core Pin	3.63	3.18	1.14	35.8
M3 Core Pin	4.75	3.56	1.33	35.8
M3.5 Core Pin	5.54	3.81	1.45	39.45
M4 Core Pin	6.38	4.7	1.35	23.71
M5 Core Pin	7.16	6.35	1.12	23.71

**Table 2:** Machine and material specification used for printing 3DIM inserts.

	MJ Machine 1	MJ Machine 2
Material	Visijet M3X	Digital ABS
Machine	Projet 3500	Object Connex 350
Manufacturer	3D Systems	Stratasys
Layer Thickness	30 Microns	30 Microns
Print Mode	Not Applicable	Matte
Cleaning	Water Jet Cleaning	Water Jet Cleaning

**Table 3:** Process parameters used for injection moulding.

Description	Value
Resin	Lexan 943-A
Manufacturer	Sabic Innovative Plastics
Type	Polycarbonate
Mould Temperature	28 °C
Melt Temperature	300 °C
Injection Pressure	60 MPa
Fill Time	0.2 Seconds
Cooling Time	45 Seconds
Mould Open Time	Open until the mould temperature returned to 28 °C

**Table 4:** Description of the variables.

Symbol	Parameter
$\sigma_{max}$	Maximum bending stress
$\Delta P_{PinSNi}$	$P_{SNF_{i}}-P_{SNB_{i}}\;$
$P_{SNF}$	Injection pressure at the first point of contact (front face)
$P_{out}$	Injection pressure at last point of contact (back face)
i	Sensor number [1,2,3,4]
*M*	Internal bending moment
*y*	Perpendicular distance from neutral axis
*I*	Moment of inertia of the section
h	Height of the core pin
d	Diameter of the pin

**Table 5:** Description of variables in shear stress formula.

Symbol	Parameter
$\tau_{max}$	Maximum shear stress
$\Delta P_{Pin}$	$P_{SNF_{i}}-P_{SNB_{i}}\;$
$P_{SNF}$	Injection pressure at the first point of contact (front face)
$P_{out}$	Injection pressure at last point of contact (back face)
i	Sensor number [1,2,3,4]

**Table 6:** Peak bending stress and shear stress on the core pin and the time at which it occurs.

Name	Diameter (mm)	Height (mm)	$\mathrm{Bending}\;\mathrm{Stress}\;\sigma_{max}\;(\mathrm{MPa})$	Shear Stress $\tau_{max}$ (MPa)	Time t^∗ (s)
M2 Core Pin	3.63	3.18	11.75	3.69	0.14
M3 Core Pin	4.75	3.56	8.65	3.16	0.14
M3.5 Core Pin	5.54	3.81	10.49	4.37	0.18
M4 Core Pin	6.38	4.7	16.77	6.33	0.08
M5 Core Pin	7.16	6.35	27.50	8.06	0.09

**Table 7:** Bending and shear stresses on the core pin with the effective diameter and yield strength of the tool material.

Name	Diameter (mm)	Height (mm)	$\mathrm{Bending}\;\mathrm{Stress}\;\sigma_{max}\;(\mathrm{MPa})$	Shear Stress $\tau_{max}$ (MPa)	Time t^∗ (s)
M2 Core Pin	2.63	3.18	20.22	3.69	0.14
M3 Core Pin	3.75	3.56	13.87	4.08	0.14
M3.5 Core Pin	4.54	3.81	15.63	5.33	0.18
M4 Core Pin	5.38	4.7	23.58	7.50	0.08
M5 Core Pin	6.16	6.35	37.15	9.37	0.09

**Table 8:** Hole depth of the M5 core pin on each of the moulded part. Red text indicates damage to the geometry.

Shot Number	M5 Hole Depth on the Part	Intended Value (mm)	Deviation (mm)
1	6.33	6.35	−0.2
2	6.33	6.35	−0.2
3	6.32	6.35	−0.3
4	6.40	6.35	+0.05
5	6.38	6.35	+0.03
6	3.89	6.35	−2.46
7	2.85	6.35	−3.5
8	2.45	6.35	−3.9
9	1.07	6.35	−5.38
10	0.98	6.35	−5.37
11	0.90	6.35	−5.45
12	0.42	6.35	−5.93

**Table 9:** Tooling comparison.

Indicative Comparison for Parts That Fit within a 100 × 100 mm Footprint	Aluminium/Steel Tooling	Polymer 3DIM Tooling	Metal 3DIM
Lead time	2 weeks	1 day	2 days
Cost of producing the tool, materials and machine costs (NZD indicative)	$500–$3000 Requires highly skilled labour	$1000 Easy to implement	$5000 Easy to produce tool, but requires specialized equipment and labour for post-processing
Dependency on complexity of geometry	HIGH: Cost and time highly dependent on complexity	NONE: Cost and time not dependent on complexity	NONE: Cost and time not dependent on complexity
Tool Life	10,000 shots Low dependency on injection material	0–100 shots High dependency on injection material	5000 shots Low dependency on injection material
Cycle time	1 s (conformal cooling is expensive to incorporate)	30 s (conformal cooling ineffective)	0.5 s (conformal cooling easy to include)

**Table 10:** Classification of raised feature failure in 3DIM.

1	Moulding Stage	Cause	Failure Mode	Status
2	Injection Stage	Flow pressure exerted by the incoming molten polymer.	Bending Failure	Current Work (Not a critical failure, unless wrong process setting used)
			Shear Failure	
3	Packing Stage	Flow pressure exerted by molten polymer and heat transfer between molten polymer and 3DIM.	Thermal degradation	
4	Cooling Stage	Heat transfer between molten polymer and 3DIM (Shrinkage of part due to cooling and expansion	Stress due to interference	Future Work
5	Ejection Stage	Surface roughness due to layered process of 3D printing. Shrinkage of part and expansion of tool. Inadequate draft angles	Edge Failure	Future Work
			Ejection Failure	

## Data Availability

Not applicable.
